# Defining dimensions of research readiness: a conceptual model for primary care research networks

**DOI:** 10.1186/s12875-014-0169-6

**Published:** 2014-11-26

**Authors:** Helen Carr, Simon de Lusignan, Harshana Liyanage, Siaw-Teng Liaw, Amanda Terry, Imran Rafi

**Affiliations:** Department of Health Care Management and Policy, Clinical Informatics and Health Outcomes Research Group, University of Surrey, Guildford, UK; School of Public Health & Community Medicine, UNSW Medicine Australia, Sydney, New South Wales 2052 Australia; Centre for Studies in Family Medicine, The Western Centre for Public Health and Family Medicine, 2nd Floor, Schulich School of Medicine & Dentistry, Western University, 1151 Richmond St, London, ON N6A 5C1 Canada; Royal College of General Practitioners, 30 Euston Square, London, NW1 2FB England

**Keywords:** General practice, Research, Medical records systems, computerised, Data collection, Patient selection

## Abstract

**Background:**

Recruitment to research studies in primary care is challenging despite widespread implementation of electronic patient record (EPR) systems which potentially make it easier to identify eligible cases.

**Methods:**

Literature review and applying the learning from a European research readiness assessment tool, the TRANSFoRm International Research Readiness instrument (TIRRE), to the context of the English NHS in order to develop a model to assess a practice’s research readiness.

**Results:**

Seven dimensions of research readiness were identified: (1) Data readiness: Is there good data quality in EPR systems; (2) Record readiness: Are EPR data able to identify eligible cases and other study data; (3) Organisational readiness: Are the health system and socio-cultural environment supportive; (4) Governance readiness: Does the study meet legal and local health system regulatory compliance; (5) Study-specific readiness; (6) Business process readiness: Are business processes tilted in favour of participation: including capacity and capability to take on extra work, financial incentives as well as intangibles such as social and intellectual capital; (7) Patient readiness: Are systems in place to recruit patients and obtain informed consent?

**Conclusions:**

The model might enable the development of interventions to increase participation in primary care-based research and become a tool to measure the progress of practice networks towards the most advanced state of readiness.

## Background

### Development of primary care research

Historically, most clinical research studies and expenditure on research were in secondary care settings rather than in primary care. A decade ago primary care research was described as a “lost cause,” [1] although this was contested at the time [2,3]. However, since then Primary Care Research Networks (PCRNs) have developed and encouraged and supported more research taking place in primary care [4-7]. Plausibly, these network have contributed to quality improvement [8]. There is some bibliometric evidence that primary care research is on the increase [9]. In England around a third of patients recruited into the National Institute for Health Research (NIHR) portfolio were for primary care-led studies. 129,000 patients participated in primary care led studies; with a total of 206,716 patients recruited into studies of all types from primary care [10].

### UK primary care as a research setting

UK general practice should be an ideal location for a wide range of research because most general practices are computerised, with a vast quantity of routine patient data recorded and potentially usable for research [11]. The UK has a registration-based system, where individual patients are registered with specific general practitioners (GPs) often for many years at a time, if not their whole lives [12,13]. Hence GPs have reasonably complete longitudinal records. A national identifier, NHS number, makes it possible to link primary and secondary care data. Linked data sources can add further value, [14] although there are challenges in data validity, security and privacy [15]. The UK’s representative body for family practice – the Royal College of General Practitioners (RCGP) - set up a Research Readiness scheme to facilitate primary care research. It saw governance as a barrier to practices delivering research and sought to provide training to facilitate approval for its scheme [16].

#### Lessons from primary care research

Many investigators view UK general practice as difficult to access and recruitment to studies as challenging. These barriers were summed up in a recent English School of Primary Care Report: [17]

*A number of publications have described the challenges associated with trial recruitment in the United Kingdom and elsewhere, some of which have even led to trials being abandoned. Up to 60*% *of trials need an extension or don’t recruit to target according to recent reviews, and concerns about this are widespread in academia and industry. Although evidence is sparse, one study of 114 UK trials in all health care contexts funded by the Medical Research Council and Health Technology Assessment programme, found that 31*% *recruited successfully, and 45*% *recruited less than 80*% *of their target. Just over half of all trials required an extension* [18]*.*

*Similar findings from a smaller survey of published primary care trials found that approximately one third recruited to timetable, one third required up to 50*% *more time than planned and another third required over 50*% *extra time than originally planned* [19]

The same picture is found internationally [20].

### Research readiness

The concept of “research readiness” is not new; it has been described for nearly thirty years [21]. It aims to explore the gap between the resources in primary care that could be used for research and what is ready and available to be used. These resources include patients, who in the UK are registered with a single practice, and their medical records. Quality improvement studies have been suggested as a halfway house in which research concepts can be introduced to health professionals and potentially lead to enhanced research readiness [22]. There are probably many factors that contribute to whether a primary care practice is ready and willing to participate in research and make its patients and their data available to researchers. One factor may be that many GPs in computerised practices are unaware of opportunities to become involved in research projects; though others are aware but not interested [23]. Part of the GP role can be as gatekeeper between primary and secondary care [24]; but also as gatekeeper between patients and researchers, thus limiting the possibility of primary care patients being recruited to studies.

However, much of what was written about research readiness predated the computerisation of primary care, which makes searching for patients who meet the criteria for inclusion in studies and the follow up of patients using the records themselves much easier [11]. We carried out this review to explore whether we could use the learning from a European project to update the concept of *“research readiness”* into a model that would help identify the key requirements for participation in research. Such a model might help PCRNs identify practices ready to participate in research and reveal how others might be brought to a state of readiness. Change in the state of readiness might also be utilised as a method of assessing the effectiveness of PCRNs.

## Method

### Overview and literature review

We conducted an evidence synthesis based on a literature review including exploration of the development of PCRNs in the UK. We focussed on identifying dimensions of research readiness or initiatives that might affect data quality or access; including the creation of national collections of primary care data.

**Figure 1:**
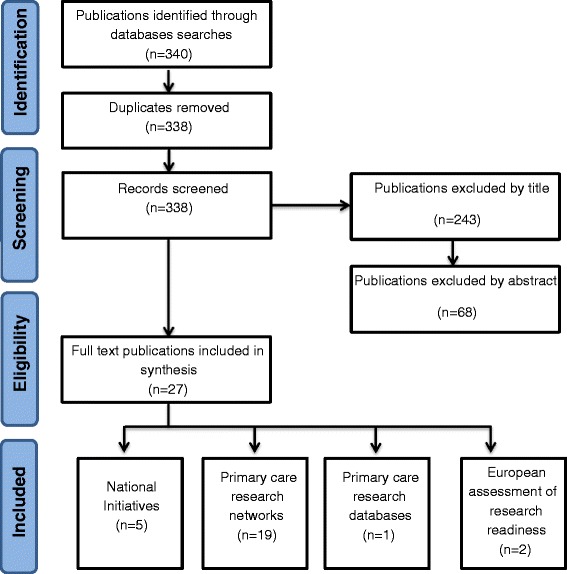
**Literature search results.**

We carried out our search using the search terms “readiness”, “research network” and “family practice” on PubMed/Medline bibliographic database. We created a Preferred Reporting Items for Systematic Reviews (PRISMA) flow diagram (Figure 1) to describe the search results. The searches identified papers between April 1976 and March 2014 search was limited to the English language. The publications from searches totalled 340. Screening process included removal of duplicates, exclusion using title and exclusion by abstract. The screening processed identified 27 relevant publications which were suitable for the synthesis. These publications were categories based on four themes: national initiatives, primary care research networks, primary care research databases and European assessment of research readiness.

### Developing a schema

Our initial theoretical approach was to consider adoption of research as a readiness to change on behalf of the practice, built on the classic model developed in the domain of smoking cessation by Prochaska and DiClemente [25]. We conceptualised this willingness to change as being provision within the practice; or the development in response to input of the PCRN of intangible resources that increased research readiness.

We explored our findings from the perspective of adding value through the development of intangible resources or assets, which we considered were an important part of becoming research ready. We analysed intangible resources in three component areas: (1) Human capital: individuals’ knowledge and know-how about getting research done; (2) Relationship capital: built up through personal contact and relationships between practices and their research network; and (3) Structural capital: the training, information systems, and other unified organisational structures provided to support primary care research [26].

Improved intangible resources are a known business strategy for improving organisational performance [27].

We developed a schema for readiness using the data that emerged from the following elements:

- The results of our literature review
- Lessons from the development of PCRNs to promote research in primary care
- Technical and other advances that facilitated access to primary care data
- Incorporating the learning from a European project which included an assessment of research readiness [28]
- Presentation and workshop at a National Primary Care meeting and the subsequent discussion groups and feedback.

### Final model development

We finally formulated a model that might be used to test research readiness. We developed a model based on the areas of intangible resources that the practices themselves, the PCRN, investigators or National initiatives might influence.

These three areas are:

1. Human capital: individuals’ know-how and commitment to fit research in;
2. Relationship capital: It is well established that strong relationships can change behaviour; and
3. Structural capital: the training, information systems, and other inputs required to improve readiness.

## Results

### English national initiatives

- Legislation and policy to promote research:
  A “top-down” approach has been used in England to improve participation in research by primary care clinicians and their practices [29]. In 2011, the Government announced funding to help develop the country’s science and research base and secure England as a world leader in health research. There is additional funding for biomedical research units to help develop medicines, treatments and care for patients with diseases such as cancer, diabetes, heart disease and dementia through a National Institute for Health Research (NIHR). The Health and Social Care Act 2012 [30]. recognised that research is a core function of the NHS and is vital to quality improvement. At primary care level, it has made the promotion of innovation and research a core duty of the work of the local NHS, with a requirement to participate in research and to promote patients’ recruitment to research. There is a strong focus on governance in the UK Department of Health approach to primary care research [31].
- Primary Care Research Networks (PCRNs)
  To date, the principal national initiative to improve primary care research capacity and capability has been the development of primary care research networks (PCRNs) [32], which have fulfilled many functions since they were established in the 1980s. Over the years their emphasis has changed from being locally-based networks which supported the research development, resourcing and interaction of local primary care teams to the current state of a more national network which encourages and supports practices to participate in large-scale clinical trials allied with the research priorities of government.

As the research conducted by PCRN-member practices includes clinical trials, all practices working within the PCRN are encouraged to undertake Good Clinical Practice (GCP) training. The EU Directive 2001/20/EC defines GCP as

*“A set of internationally-recognised ethical and scientific quality requirements which must be observed for designing, conducting, recording and reporting clinical trials that involve the participation of human subjects. Compliance with this good practice provides assurance that the rights, safety and well-being of trial subjects are protected, and that the results of the clinical trials are credible and accurate”* [33]*.*

PCRNs have also had other functions, including promoting research networking events and developing research governance within primary care. In an age of computerised medical records this includes information governance and data protection. This latter is important as the practice cannot delegate its responsibility to its patients and must safeguard their data; the practice is by data protection statute the “Data controller” [34].

Perhaps less well-documented is the value of the relationship capital and other intangible resources built up between PCRNs and practices in their networks. We only identified one review that considered intangible resources; it describes how social and intellectual capital are the two key resources developed by PCRNs [35]. This was reinforced during discussions at a National Primary Care Research Networks conference at which there was recognition of the extraordinary value of the social (i.e. relationship) capital, intellectual capital and general know-how built up within the PCRN but acknowledgment that this was not articulated through any particular intellectual frame. Whilst Fenton et al. had reflected on organisational issues and recognised their importance in their paper, they excluded microeconomics from their analysis. Other countries are at an earlier stage of developing primary care research networks, with Practice Based Research Networks (PBRNs) in Australia [36] and the Canadian Primary Care Sentinel Surveillance Network being similar models [8,37].

c) Royal College of General Practitioners - Research Ready Scheme

“Research Ready” is an online tool developed for practices by the UK Royal College of General Practitioners (RCGP) and the PCRN. It covers the basic practicalities for conducting research and describes five core competencies. The first three describe the human resource, physical space and ability to run computer searches to identify patients or extract data for research. The final two address the minimum requirements of the Research Governance Framework for undertaking primary care research in the UK [38]. The RCGP, in collaboration with the National Institute for Health Research, is currently updating and refining the way in which practices are accredited to the scheme, developing online training modules for practice staff with the aspiration of developing a leadership role amongst other practices less experienced in research.

### IT developments that facilitate primary care involvement in research

- Standardising primary care computer systems:
  General practice has proved easier to computerise than hospital care. One of the contributors to this in the UK was a Requirement for Accreditation of GP computer systems, which ensured there was a degree of standardisation between the systems. The standardisation included the coding system used to record diagnoses, symptoms, investigation results and treatment [39]; Read codes have been used in the NHS since 1985. The use of EPR systems combined with standardised coding systems within them, is now almost universally used at the point of care; computerised primary care EPR data have enormous potential for research [40].
- Making primary care health data accessible for research:

There are three primary ways that primary care computer system data have been made more accessible for research:

- Search tools provided by the vendor allow case-finding within a practice
- In the UK a standard extract tool was created, allowing the same search to be run across different vendors’ systems. In the UK the first such tool to be developed was Morbidity Information and Export Syntax (MIQUEST) [41].
- Creation of research databases initially based on single computer systems. These data also have shortcomings; [42] and extraction methods have imperfections but the types of errors can be classified to enable a rational approach towards addressing them [43].
- Newer UK developments in 2013 include the establishment of the Farr Institute of Health Informatics Research [44] which aims to link electronic health data, including primary care data, with other forms of research and population data.
- Attempt to start to build up groups of patients who individually consent to their data being used and can be contacted directly to participate in research. An example of this is the Scottish SHARE [45] initiative which aims to establish a register of people interested in participating in health research.

The oldest network or research database is that established by the Royal College of General Practitioners (RCGP) to collect morbidity data and carry out surveillance for infectious diseases and conduct research into vaccine effectiveness [46]. This is now known as the RCGP Research and Surveillance Centre (RSC) [47]. Not all research databases survive; the Doctors Independent Network Database (DIN) [48] has now closed, as has the computer systems it was based upon. There have been other systems established around single practice electronic patient record (EPR) systems, for example:

- Clinical Practice Research Datalink (CPRD) which started in a single EPR system but has now been extended to include other brands, [49,50]
- QResearch is based on the EMIS (Egton Medical Information System), [51]
- The Health Improvement Network (THIN), [52,53]
- ResearchOne [54].

The Clinical Practice Research Datalink (CPRD) is probably the most successful database in terms of research output. It is an observational data and interventional research service which has been built upon the General Practice Research Database (GPRD) [55]. The GPRD contains anonymised health data pertaining to consultations, prescriptions, referrals and health outcomes. The data is collected from a particular GP computer system named “Vision” and includes a built-in data collection component. When forming the CPRD, the GPRD was combined with the Health Research Support Service (HRSS), a program developed to help researchers access and analyse health care and relevant data to support their research projects. As CPRD it has plans to expand further and to have national coverage.

### Learning from a European project which included an assessment of research readiness

A European project (TRANSFoRm) aimed to facilitate research by linking together data from primary care databases with either genetic or disease registry data, and resulted in the development of a survey instrument (TIRRE) designed to assess readiness to participate in such linked research [56,57].

The TIRRE survey instrument from the TRANSFoRm project initially identified four main areas of readiness: data readiness, record readiness, organisational readiness (including health system structure and socio-cultural factors) and study-specific readiness (Table 1). However, after further analysis it became clear that business process readiness, which includes the capacity and capability to take on extra work, was also important [58].

**Table 1:** **TIRRE dimensions of research readiness**

**TIRRE model of research readiness**	
**1**	**Data readiness (micro level)**
This will assess the current state of data held within the practice.	
a	What data
	i. Scope of data recorded
	ii. How held (distributed or centralised)
	iii. Single or multiple systems
b	Interoperability
	i. Denominator data, - demographics, - unique identifiers
	ii. Coding system
	iii. Data quality – metadata Linkages – lab
**2**	**Record system readiness (meso level)**
a	Type of record architecture – encounter based, problem orientated,
b	Data extraction method (e.g. local or central)
c	Extract type
d	Health-system-wide initiatives for data extraction (e.g. CPRD, GPES)
**3**	**Organisational readiness (macro level)**
a	Legislative and regulatory compliance readiness
b	Health system readiness
	i. Organisational structure
	ii. Local issues or service configuration that might inform data availability
	iii. Other studies which may involve the target patients/subjects of research
c	Socio-cultural readiness
	i. Types of studies that the data provider finds acceptable/is allowed to participate in
	ii. Other factors that might influence local data
	iii. Language within records
**4**	**Study readiness**
a	Quality of relevant data
b	Demographic and other data including access to laboratory and imaging res

We combined the learning from the TIRRE project with the existing Royal College of General Practitioners (RCGP) Research Ready scheme to develop a new readiness model which would assess whether GP practices were truly research-ready, and would provide investigators with a mechanism to assess research readiness in potential participants.

#### Final research readiness model

**Figure 2:**
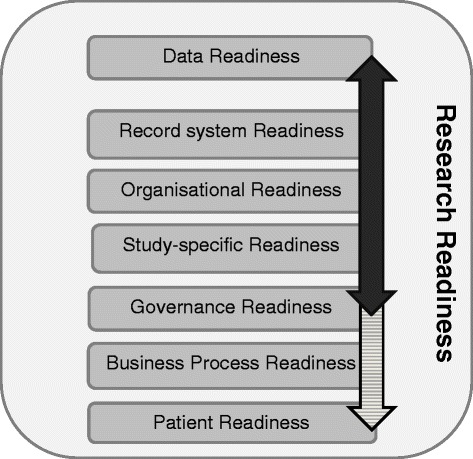
**Dimensions of research readiness.** Bold arrow – TIRRE model, shaded arrow extended model.

The “dimensions of research readiness” model specifies seven perspectives from which a practice might be assessed in order to determine its readiness to participate in research (Figure 2, Table 2). Including a “study specific” requirement enables researchers to set out any research requirements not covered elsewhere in the readiness model. The business process, including workload, has to work if practices are to participate in studies; and whilst this aspect of readiness is modulated by the type of study, in the end most practices require reimbursement for the time taken to participate in research. Patients also need to be “ready” to participate in research; the authors’ experiential learning is that it is more difficult to recruit in research-naïve practices than in those experienced in research. The other new dimension is that of “governance readiness”. This has been moved out of its previous position within organisational readiness to be a dimension in its own right, acknowledging the enormous increase in its prominence since development of the original TIRRE model.

**Table 2:** **New Model of dimensions of readiness of practices to participate in research**

	**Dimension of readiness**	**Key attribute(s)**	**Health System & Research network activity to promote research readiness**	
			**Existing**	**New activity required**
**1**	**Data**	Coded data that identifies:	Pay-for-performance (P4P) has improved (but also distorted) data quality	Active engagement in data quality (of cases & likely controls)
		Denominator		
		Cases (& controls)		
		Inclusion & exclusion criteria		
**2**	**Records**	Data are extractable	Networks that extract data (research databases)	Validation of extracts is required: these can have errors and be inconsistent.
			One-off (MIQUEST) extraction	
			Practice searches (EPR vendor search tool)	
**3**	**Organisational**	Health system readiness	Legislation (Health & Social Care Act 2012)	Engagement with local primary care structures (Health service localities; Medical primary care societies etc.)
		Socio-cultural	Government/Health ministry promotion of bioscience research	
			Incentive schemes for practices	
**4**	**Governance**	Research governance (RG)	RG emphasis of existing scheme	Educational programme
			Good Clinical Practice (for trials)	
		Information governance		
			Some confusion about “Opt out”	
			Practice has legal responsibility as the *Data Controller* in the UK (Data Protection Act)	New national guidance about personal data is required.
**5**	**Study**	Impossible to cover all eventualities	Data quality for the specific study	Responsive support, direct data collection from patients may be possible
			Demographic data	
**6**	**Business**	Tipped in favour or participation	Mechanism for funding research (e.g. some practices reluctant to carry out studies sponsored by pharmaceutical industry)	Standard payments
				Use quality improvement studies to promote research-relevant activities
			Level of funding and whether provides sufficient incentive to participants	Develop intangible resources
				(social/relationship capital)
			Feasibility of study being incorporated into existing workload	
			Any risk/perceived risk (e.g. new drug)	
**7**	**Patient**	Information consent	Individual expectation to participate in research/“pre-consent” models	Learn how to take consent
				Develop intangible resources (relationships with practices)
			Volunteer patient cohorts	
			Single disease (e.g. diabetes), where there may be an associated primary care clinic	
			Patient-practice culture & ethos about participating in research	
			Track record – previous experience of delivering projects - type, clinical domain, number of cases	

## Discussion

### Principal findings

Current models of readiness are limited and there is scope for development. Whilst the TIRRE instrument provided insight into the breadth and depth of research readiness, we have had to develop the model further, formalising the need to include business process modelling and identifying that intangible resources are also important. We have added culture, ethos and track record (of research participation), and recognised there will often be additional study-specific requirements.

We propose this seven-element model is used to explore research readiness and to assess the suitability of projects to recruit successfully in primary care Figure 2.

### Implications of the findings

Research readiness models should be broad and multifaceted if they are to fully address the requirements for effective involvement in research. This new model could become the basis of the next iteration of TIRRE or another tool to assess the readiness of practices to participate in primary care research. Areas for further development are to optimise approaches that achieve the “buy-in” of busy clinicians working in ordinary primary care settings without any formal links to academic institutions. These areas represent important intangible assets that need to be developed; particularly human and relationship capital [59].

### Comparison with the literature

The report from the English School of Primary Care provides a comprehensive list of strategies for researchers, but is written more from the perspective of overcoming barriers to recruitment [17]. A Cochrane review looked at strategies to improve recruitment of participants to randomised controlled trials. The review suggested that telephone reminders, opt-out rather than opt-in, and open designs where patients know their treatment all help; however it did not report evidence of organisational readiness being a key factor [60]. An Australian study around managing clinical data requirements for a compulsory national performance system defined the key features of “readiness” as staff skills, supportive management and a high level of trust from participating practices [61]. An American editorial commented that attempts to define research capacity within primary care, let alone readiness, have been incomplete although there is encouraging evidence of enthusiasm [62]. Another American study found that the key incentives for primary care doctors to collaborate with academic researchers were the potential to enact quality improvement, make a contribution to the body of knowledge, and intellectual stimulation [63]. An editorial focussed on the necessity of collaboration and sharing of expertise and resources; [64] and a further Australian report argues that it is important that network are properly resourced, based in academic departments, and that there is more interventional research carried out on a larger scale [36].

Finally, there is much written on the importance of intangible resources or assets in industries that rely on relationships and communication to get things done, though relatively little in health care. [65] PCRNs have yet to document the extent to which they have created social and intellectual capital, which might soon be lost as they are amalgamated into comprehensive research networks. The case for investment in structural capital, particularly better use of informatics, has been made on the basis that this may be one of the biggest bars to recruitment [66]. A Canadian series on research networks emphasises the central role of the EPR in research [67]. Developing research in primary care is also seen as a tool for developing primary care per se [8,68].

### Limitations of the method

The limitation of the method is the lack of literature about the barriers to conducting research in primary care and how to overcome them, and why even a practice that appears to be “research-ready” may choose not to participate in a given study. Possibly major research bodies should start to have a common format for recording delays in recruitment and analysing them against dimensions of readiness, to develop a greater understanding and evidence base.

### Call for further research

It would be possible to develop a modified version of the TIRRE instrument to assess research readiness quantitatively. Such a readiness score could be compared with practices’ participation in different types of studies. The information about the study would include its type e.g. cross-sectional, cohort, trial; and the level of involvement of patients. Observational studies, for example, can have a considerable data-recording burden; willingness to participate in interventional studies may be influenced by whether the intervention is a pharmaceutical or operative one, compared with quality improvement or psychological interventions. Whether the funder is a research institute or from industry may also influence uptake. The readiness score will provide a basis for measuring improvement in readiness and for evaluating what might be predictive factors of willingness to participate in research. Readiness scores may also be useful in identifying which dimensions are more challenging to improve.

## Conclusions

A new broader approach to research readiness might help standardise recording of readiness and articulate the reasons why practices do or don’t participate in particular research studies. This study’s limitation is that the model has been developed based on experiential learning and a very limited evidence base. However, it is plausible that such a model might enable the development of interventions to raise participation in primary care-based research and become a tool to measure PCRN performance in terms of the numbers of practices they bring forward to the most advanced state of readiness. A more sophisticated approach towards developing practices’ levels of research readiness may help address issues with recruitment into research studies in primary care.

## Abbreviations

EPR: Electronic Patient Record
TIRRE: TRANSFoRm International Research Readiness instrument
PCRN: Primary Care Research Network
NIHR: National Institute of Health Research
GP: General Practitioner
RCGP: Royal College of General Practitioners
GCP: Good Clinical Practice
CPRD: Clinical Practice Research Datalink
GPRD: General Practice Research Database
